# Antibacterial and pH-sensitive methacrylate poly-L-Arginine/poly (β-amino ester) polymer for soft tissue engineering

**DOI:** 10.1007/s10856-023-06720-8

**Published:** 2023-04-10

**Authors:** Parisa Heydari, Jaleh Varshosaz, Mahshid Kharaziha, Shaghayegh Haghjooy Javanmard

**Affiliations:** 1 Department of Materials Engineering, Isfahan University of Technology, Isfahan, 84156-83111 Iran; 2 Applied Physiology Research Center, Isfahan, Iran; 3 Novel Drug Delivery Systems Research Center, Department of Pharmaceutics, School of Pharmacy and Pharmaceutical Science, Isfahan University of Medical Science, Isfahan, Iran; 4 Cardiovascular Research Institute, Isfahan University of Medical Sciences, Isfahan, Iran

**Keywords:** Methacrylate poly-L-Arginine, Poly (β- amino ester), pH-sensitive polymer, antibacterial properties

## Abstract

During the last decade, pH-sensitive biomaterials containing antibacterial agents have grown exponentially in soft tissue engineering. The aim of this study is to synthesize a biodegradable pH sensitive and antibacterial hydrogel with adjustable mechanical and physical properties for soft tissue engineering. This biodegradable copolymer hydrogel was made of Poly-L-Arginine methacrylate (Poly-L-ArgMA) and different poly (β- amino ester) (PβAE) polymers. PβAE was prepared with four different diacrylate/diamine monomers including; 1.1:1 (PβAE1), 1.5:1 (PβAE1.5), 2:1 (PβAE2), and 3:1 (PβAE3), which was UV cross-linked using dimethoxy phenyl-acetophenone agent. These PβAE were then used for preparation of Poly-L-ArgMA/PβAE polymers and revealed a tunable swelling ratio, depending on the pH conditions. Noticeably, the swelling ratio increased by 1.5 times when the pH decreased from 7.4 to 5.6 in the Poly-L-ArgMA/PβAE1.5 sample. Also, the controllable degradation rate and different mechanical properties were obtained, depending on the PβAE monomer ratio. Noticeably, the tensile strength of the PβAE hydrogel increased from 0.10 ± 0.04 MPa to 2.42 ± 0.3 MPa, when the acrylate/diamine monomer molar ratio increased from 1.1:1 to 3:1. In addition, Poly-L-ArgMA/PβAE samples significantly improved L929 cell viability, attachment and proliferation. Poly-L-ArgMA also enhanced the antibacterial activities of PβAE against both *Escherichia coli* (~5.1 times) and *Staphylococcus aureus* (~2.7 times). In summary, the antibacterial and pH-sensitive Poly-L-ArgMA/PβAE1.5 with suitable mechanical, degradation and biological properties could be an appropriate candidate for soft tissue engineering, specifically wound healing applications.

**Graphical Abstract:**

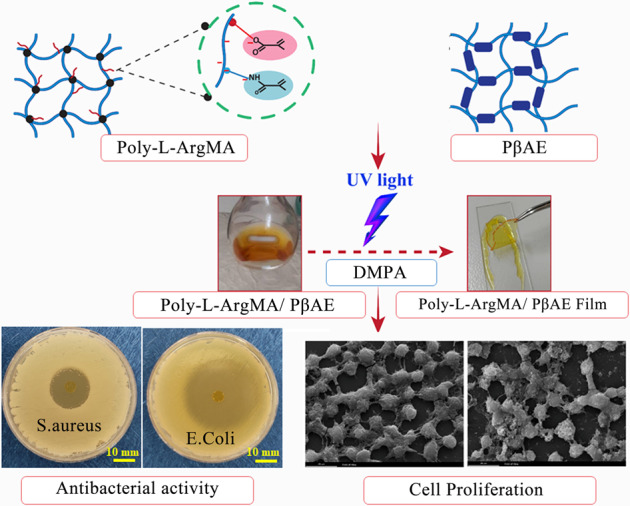

## Introduction

Soft tissue engineering combines biological science and engineering principles to improve regeneration of soft tissue, and has recently emerged as an interdisciplinary field [1, 2]. Biomaterials should have mechanical properties near the tissue and should be degradable at a controllable rate, according to the rate of target tissue regeneration [3–5]. Other specific properties of biomaterials applied for soft tissue engineering are the ability to control drug release molecule and antibacterial properties to overcome bacterial infection and accelerate tissue regeneration [6, 7]. The physicochemical properties of synthetic polymers can be adjusted by changing the synthesis process or optimizing their chemical structure [8, 9]. Among various biodegradable cationic polymers, poly (β-amino ester) (PβAE)s are synthetic polymers with a wide range of biodegradability, swelling ratio, and mechanical performances. PβAE is often synthesized using the aza-Michael polyaddition reaction between diacrylate and diamine [10, 11]. Numerous chemical structures of PβAEs could be easily obtained through changing the reactive amine or diacrylate components or their ratio leading to adjustable degradation time from hours to months and the mechanical properties such as tensile strength in a wide range of 0.1–300 MPa [10, 12]. In addition, the ionization of the tertiary amines in the PβAE backbone at a relatively pH (˂6.5) acts as a hydrophilic material and gets to be as a hydrophobic polymer by deionization of tertiary amines at higher pH (˃6.5) [13, 14]. This pH-sensitive property of PβAE could be easily adjusted via varying the monomer composition and ratio. In the last decade, PβAEs have been successfully used in gene delivery [15], drug delivery [16, 17], controllable bioactive agent release [18], and tissue engineering [19]. Despite the appropriate biocompatibility and adjustable physical and mechanical properties, PβAE often blended with other drugs, or polymers to optimize its biological properties such as antibacterial properties and cell/biomaterial interaction. For instance, Xu et al. [9] developed injectable PβAE/thiolated hyaluronic acid hydrogels with different degradation profiles (3–15 days) to promote the wound healing process and found the mixture of poly(ethylene glycol) diacrylate and Ethylenediamine monomers could adjust PβAE molecular weight in the range of 2–10 kDa. In addition, the side chains of PβAEs can be easily conjugated with methacrylate and carboxylic acid-containing compounds via esterification/amidation which could be effective for copolymerization with other polymers or biological agents to develop desired properties for tissue engineering [20, 21].

Poly-L-Arginine (Poly-L-Arg) is one of the polypeptides that has been widely applied in the mixture with synthetic polymers to improve their biocompatibility, antibacterial activity and biological properties [22]. Poly-L-Arg has a well-known function for the regeneration of damaged tissues in two main ways; First, arginase leads to proline production, which is a vital amino acid for promoting collagen synthesis. Second, the oxidative pathway controls the nitric oxide (NO) production, which is important for enhancing epithelialization, angiogenesis, and immune response activities [23, 24]. Bygd et al. [22] studied the biological ability of Poly-L-Arg for adjusting fibroblast cell responses. Their results demonstrated that the Poly-L-Arg increased the VEGF secretion, collagen fiber orientation and cell migration, leading to the acceleration of wound healing process. Amirnejat et al. [25] also synthesized nanoparticles coated with Poly-L-Arg and grafted alginate for biomedical applications. They indicated high antibacterial activity of the alginate/Poly-L-Arg composite against Gram-positive and Gram-negative bacteria after adding Poly-L-Arg.

Inspired by the advantages of PβAE as a building block and Poly-L-Arg in damaged tissue regeneration, in this study, a novel antibacterial and pH-sensitive polymers based on methacrylate poly-L-Arginine/poly (β-amino ester) with adjustable chemical and mechanical properties are synthesized for tissue engineering application. In this regard, after the synthesis of Poly-L-Arg, methacrylate Poly-L-Arg is synthesized to develop UV-cross-linkable methacrylate poly-L-Arginine/poly (β-amino ester) copolymers using PβAEs with different monomer ratios. Moreover, the role of various PβAE monomers with different molar ratios of diacrylate: diamine (1.1:1, 1.5:1, 2:1, 3:1) and methacrylate poly-L-Arginine/poly (β-amino ester) samples on the physical, chemical, mechanical and biological properties of the final polymers are evaluated.

## Materials and methods

### Materials

L-Arginine, 1-ethyl-3-(3-(dimethylamine) propyl) carbo diimide hydrochloride (EDC, ≥98%), 2-(N-morpholino)-ethane sulfonic acid (MES, >99 %), N-hydroxysuccinimide (NHS), ethanolamine hydrochloride (ETA, ≥99%), 2,2-dimethoxy-2-phenyl-acetophenone (DMPA), methacrylic anhydride (MA) (C_8_H_10_O_3_), 3-(4,5-dimethylthiazol-2-yl)-2,5-diphenyltetrazolium bromide (MTT), and Dialysis membrane (Mol. Wt. cut-off 1 kDa) were purchased from Sigma-Aldrich (USA). 1,4-Butanediol diacrylate, N, N-dimethyl ethylene diamine, dichloromethane (DCM), diethyl ether, and dimethyl sulfoxide (DMSO) were purchased from Merck (Germany). Also, Dulbecco’s Modified Eagle Medium (DMEM-high), fetal bovine serum (FBS), streptomycin, and penicillin were purchased from Bioidea, Iran.

### Synthesis of Poly-L-ArgMA

Poly-L-Arg was first synthesized, according to Carvalho et al. [26] study, with some modifications. Briefly, after preparation of L-Arg (590 mg) solution in 28 mL of 0.25 M MES buffer with pH 5.5 ± 0.2, EDC (2.99 g) was added to it and mixed for 3 h. Following the addition of ETA(4.48 g) to stop the chemical reaction, the solution was dialyzed with a 1000 Da cutoff cellulose dialysis membrane for 48 h against distilled water (DDW) at 40 ± 1 °C.

Methacrylate Poly-L-Arg was synthesized using a simple reaction between Poly-L-Arg and MA. Following the preparation of 10 wt.% Poly-L-Arg in 10 ml of DDW at 50 °C, MA (4.2 mL) was slowly added to it with a constant rate of 0.2 mL/min and was allowed to react for 2 h at 50 ± 3 °C. Consequently, the final solution was dialyzed for 7 days at 45 °C using a 1000 Da cutoff cellulose dialysis membrane to remove unreacted MA. Poly-L-ArgMA solution was refined and then lyophilized after being frozen at −50 °C. The lyophilized Poly-L-ArgMA was kept at −15 °C for the next experiments.

### Synthesis of poly (β- amino ester)

PβAE polymers were synthesized via a simple reaction between a diacrylate monomer (1,4-butanediol diacrylate) and a primary diamine (N, N-dimethyl ethylene diamine) at 50 °C through Michael addition reaction, according to a previous study with minor modification [27]. The diacrylate: diamine monomer molar ratios were adjusted to 1.1:1, 1.5:1, 2:1, and 3:1. Following the mixing the desired amount of diacrylate with DCM, the diamine monomer was combined to this solution, and mixed for 48 h at 50 °C, when a viscous yellow solution was obtained. Consequently, the solution was precipitated into diethyl ether to remove unreacted monomers such as a residual amine. As prepared PβAEs were stored at 4 °C until the next experiments. Different PβAEs were prepared by varying the acrylate to amine molar ratio (1.1:1, 1.5:1, 2:1, 3:1), named PβAE1, PβAE 1.5, PβAE 2, and PβAE 3, respectively.

### Synthesis of Poly-L-ArgMA/PβAE

Poly-L-ArgMA/PβAE with different grades of PβAE were designed via a 1:1 weight ratio of Poly-L-ArgMA and PβAE polymers. Primarily, 1 wt.% aqueous solution of Poly-L-ArgMA was prepared at room temperature and mixed with PβAEs. Consequently, EDC and NHS were mixed with the polymer solution and stirred at 4 °C for 4 h. In our system, EDC: NHS: (PβAE/Poly-L-ArgMA) mass ratio was fixed at 1:1:12. Finally, the solution was dialyzed against DDW for 4 h at room temperature. By using a 10 kDa cutoff cellulose dialysis membrane to remove the residue of EDC and NHS.

Biodegradable Poly-L-ArgMA/PβAE hydrogels were subsequently developed using a UV-initiated polymerization process. In this part, 10 wt.% Poly-L-ArgMA/PβAE solutions in 1 mL of DCM was prepared and mixed with 1 wt.% DMPA UV-initiator. After 60 s vortexing, the mixture was added to glass plates and exposed to irradiation of UV-A (1.69 mW/cm^2^) for 10 min. After the cross-linking process, the hydrogel was removed from the glass, washed for 15 min to remove any unreacted initiator, and then lyophilized. PβAE was similarly prepared for the following characterizations.

### Characterization of Poly-L-ArgMA/PβAE

#### Physicochemical characterization

To study the chemical structure of Poly-L-Arg, Poly-L-ArgMA, PβAE homopolymers, copolymers, and their hydrogels, Fourier transforms infrared (FTIR) spectroscopy (Tensor, Bruker, Germany) was used, in the range of 400–4000 cm^−1^. Furthermore, the chemical modification of Poly L-ArgMA was studied by 1H NMR spectroscopy (Bruker, 500 MHz). 1H NMR spectra of Poly-L-Arg and Poly-L-ArgMA were studied in D_2_O solvent at 26 °C. Moreover, the molecular weight of the synthesized polymers and their copolymers was verified by gel-permeation chromatography (GPC, Knauer, Germany). While the powder of L-Arg and Poly-L-Arg were dissolved in DDW, PβAE homopolymers and copolymers were dissolved in tetrahydrofuran (THF) for NMR spectroscopy. Also, the zeta potential of the homopolymers and copolymers was determined by Horiba SZ-100 (Japan). The samples were dissolved in DDW and homogenized by an ultrasonic probe (Hielscher, UP400Sfor, Germany) for 30 min and analyzed using a zeta sizer equipped with DLS (Dynamic Light Scattering) device. The wettability the films was also estimated using water contact angle evolution at room temperature (*n* = 3).

#### In vitro degradation and swelling ratio evolution

To study the role of Poly-L-ArgMA on the mass swelling ratio (MSR) and stability of the PβAE samples, PβAE with different molar ratios of monomers, and their copolymers (*n* = 3) were freeze-dried, weighted (W_1_), and soaked in 5 ml of PBS solution at pH = 5.6 and 7.4 at 37 °C for 1 h. The wet films were weighed (W_2_), and the water swelling ratio was calculated according to Eq. (1) [28].

1 $${{{\mathrm{Swelling}}}}\,{{{\mathrm{ratio}}}}\,\left( {{{\mathrm{\% }}}} \right) = \frac{{{{{\mathrm{W}}}}_2 - {{{\mathrm{W}}}}_1}}{{{{{\mathrm{W}}}}_1}} \times 100$$

Moreover, to calculate the degradation rate of samples, the films were dried, weighed (W_1_), and soaked in 5 ml of PBS solution at pH 5.6 and 7.4 (*n* = 3). After 1, 3, 7, 14, and 21 days of incubation at 37 °C, the samples were dried and were weighed (W_2_). Finally, the degradation degree was measured according to Eq. (2) [29].

2 $${{{\mathrm{Degree}}}}\,{{{\mathrm{of}}}}\,{{{\mathrm{degradation}}}}\,\left( {{{\mathrm{\% }}}} \right) = \frac{{{{{\mathrm{W}}}}_1 - {{{\mathrm{W}}}}_2}}{{{{{\mathrm{W}}}}_1}} \times 100$$

#### Mechanical properties evaluation

Mechanical properties of Poly-L-ArgMA/PβAE and PβAE films were characterized by using a tensile assay (Hounsfield, load cell 500 N, United Kingdom) and load cells with 500 N capacity, according to ASTM D2990 standard. The dry films with dimension of 30 mm × 10 mm × 0.5 mm (*n* = 6) were cut, according to the previous study [30]. The strain rate was adjusted at 1 mm/min. Consequently, according to the stress-strain curves, tensile strength, percentage of elongation, toughness (the area under the stress-strain curve), and elastic modulus (the gradient of stress-strain curve in the linear area) were estimated.

### In vitro cell viability

To investigate the role of Poly-L-ArgMA and different PβAEs on cellular interaction, the L929 fibroblasts, purchased from Iran National Cell Bank, was used. First, the hydrogels with a 10 mm diameter were prepared and sterilized using 30 min soaking in 75 v/v% ethanol and 1 h UV light exposure. L929 cells were incubated in DMEM containing 10 v/v% FBS and 1 v/v% streptomycin/penicillin at 5% CO_2_ and 37 °C. Consequently, 10^4^ cells/well of L929 were seeded on tissue culture plate (TCP) (control) and different films. During 5 days’ incubation, the following tests were performed:

The cell viability was evaluated by using an MTT assay. After 1, 3 and 5 days, the culture medium was discarded and 100 μL MTT solution (5 mg/ml) was incubated with the cell-seeded samples and control for 3 h. Consequently, after the dissolution of the dark blue formazan crystals in DMSO, 100 μL dissolved formazan solution of each sample was transferred to a 96-well plate and the optical density (OD) was evaluated with a microplate reader (BioRad, USA) against DMSO at a wavelength of 490 nm. The relative cell survival was calculated based on the following equation [31]:

3 $${{{\mathrm{Relative}}}}\,{{{\mathrm{cell}}}}\,{{{\mathrm{survival}}}}\,\left( {{{{\mathrm{\% }}}}\,{{{\mathrm{to}}}}\,{{{\mathrm{control}}}}} \right) = \frac{{{{{\mathrm{X}}}}_{{{\mathrm{S}}}} - {{{\mathrm{X}}}}_d}}{{{{{\mathrm{X}}}}_{{{\mathrm{t}}}} - {{{\mathrm{X}}}}_{{{\mathrm{d}}}}}}$$

Where X_S_, X_d_, and X_t_ were OD of the sample, DMSO as the blank sample, and TCP as the control group, respectively.

To investigate the L929 cell adhesion and spreading on Poly-L-ArgMA/PβAEs and PβAEs hydrogels, after 3 days of cell seeding the samples were rinsed with PBS and fixed with 1.5% glutaraldehyde for 4 h at 4 °C. Then, the cells were dehydrated with the graded concentrations of ethanol and air-dried. Before SEM imaging, the samples were gold-sputtered in a vacuum and viewed using Scanning Electron Microscopy (SEM, Philips) [32, 33].

### In vitro antibacterial activity evaluation

The antibacterial properties of films were investigated against Gram-negative *Escherichia coli* (*E. coli*) and Gram-positive *Staphylococcus aureus* (*S. aureus*) bacteria. In this regard, Poly-L-ArgMA/PβAEs and PβAEs hydrogels were cut with a 7 mm diameter and placed into a nutrient Hinton agar environment with *S. aureus* and *E. coli* suspension on separate petri dishes at 37 °C. The antibacterial activity was studied after 24 h according to the zone of inhibition for each group [34]. To evaluate antibacterial activity and bacterial adhesion, Scanning Electron Microscopy (SEM) was used. The sterilized hydrogels were incubated at 37 °C for 24 h. After incubation, samples were washed carefully with sterilized water and fixed with 2.5 v/v% glutaraldehyde for 1 h. Then, the bacteria were dehydrated with the graded concentrations of ethanol (30, 40, 50, 75 and 96%) and air-dried. Before SEM imaging, the samples were gold-sputtered in a vacuum and viewed using SEM (Philips) [35].

### Statistical analysis

In this study, all data were analyzed by using a one-way ANOVA test. For evaluation the statistical significance between groups, Tukey–Kramer post hoc test using Graph Pad Prism Software (V.9) was applied and *P* value < 0.05 was defined as statistically significant.

## Results and discussion

### Synthesis and characterization of Poly-L-ArgMA

**Fig. 1:**
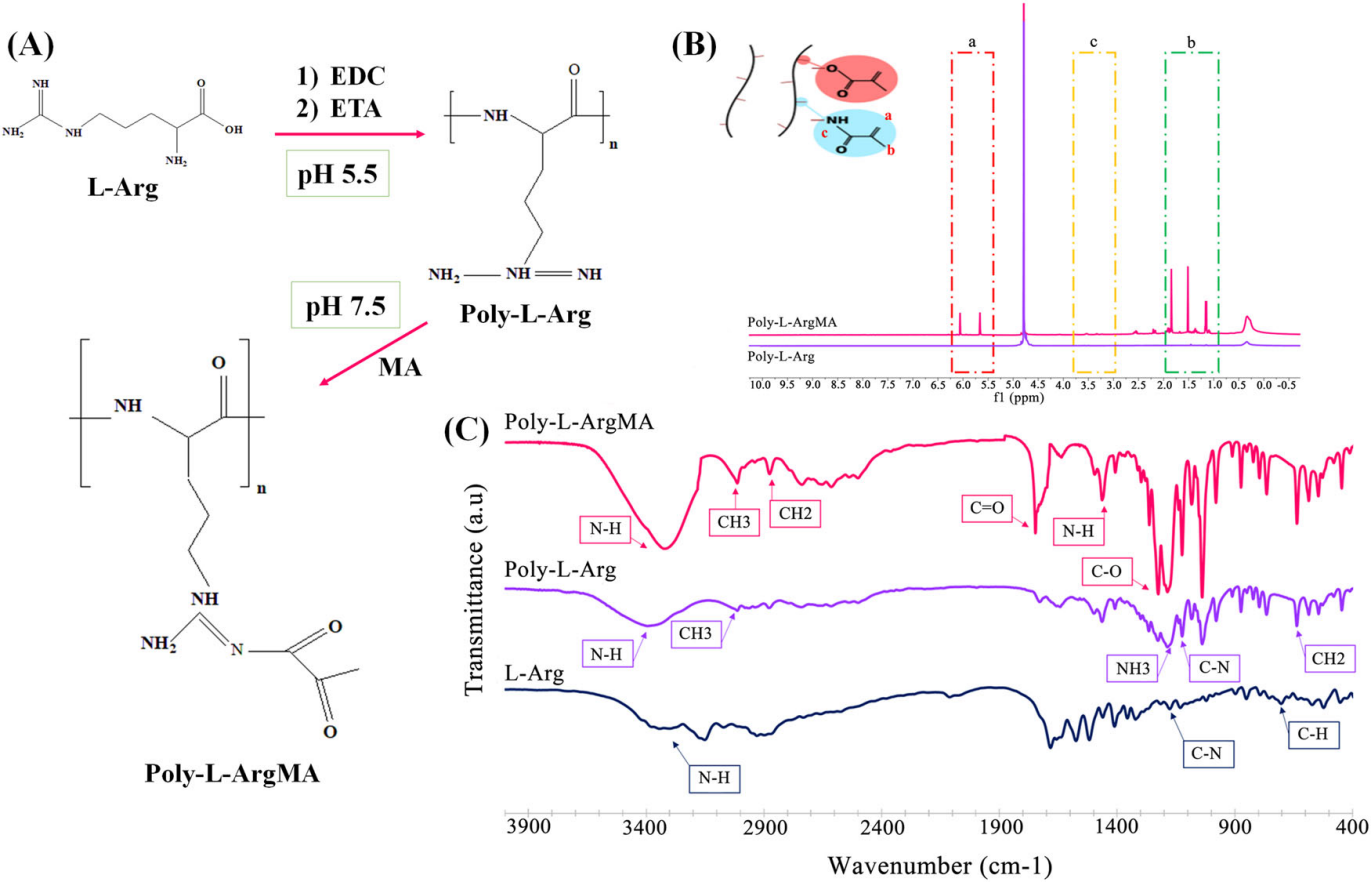
Characterization of methacrylate Poly-L-Arg: **A** The chemical structure schematic of the synthesis and methacrylation of Poly-L-Arg, **B** 1H NMR spectra of Poly-L-Arg and Poly-L-ArgMA, and **C** FTIR spectra of Poly-L-ArgMA, compared to L-Arg and Poly-L-Arg

In this study, we introduced a new antibacterial and pH-sensitive Poly-L-ArgMA/PβAE for tissue engineering application. At first, Poly-L-Arg polymer was synthesized and consequently was modified using methacrylation process (Fig. 1A). The methacrylation process of Poly-L-Arg was evaluated using ^1^H NMR spectroscopy (Fig. 1B). The spectrum of Poly-L-ArgMA consisted of the methyl group at δ = 1–1.8 ppm, the protons of methylene groups at δ = 3.3 ppm, and methacrylate vinyl group at 5.5–6 ppm. These peaks were similarly reported during the methacrylation of gelatin (GelMA), albumin, and Kappa-carrageenan [36–38]. In addition, the methacrylation degree of Poly-L-ArgMA was also estimated at 46.2 ± 8%, according to the difference between the surface below the two ^1^H NMR spectra of Poly-L-Arg and Poly-L-ArgMA (Fig. 1A). The FTIR spectrum in Fig. 1C demonstrated the successful polymerization and methacrylation of Arg. L-Arg monomer consisted of a C–H bond at 703 cm^−1^, C–N bond at 1174 cm^−1^, and a stretching of the amine group at 3100 cm^−1^ [39]. The spectrum of Poly-L-Arg also showed numerous peaks in the range of 600–3000 cm^−1^, which could be defined as the various chemical groups, such as CH_2_ at 636 cm^−1^, C–N at 1122 cm^−1^, NH_3_ at 1639 cm^−1^, CH_3_ at 3014 cm^−1^, and N–H at 3380 cm^−1^ [26]. After methacrylation of Poly-L-Arg, the new peaks were identified, including the C–O bond at 1224 cm^−1^, the N–H group at 1463 cm^−1^, and the C=O at 1747 cm^−1^, and the intensity of N–H peak at 3300 cm^−1^ increased. Our results demonstrated the successful polymerization of Poly-L-Arg and the addition of C–O, N–H and C=O functional groups demonstrated methacrylation of this polymer. This result confirmed the successful methacrylation of Poly-L-Arg for the first time. In addition, the methacrylation degree of Poly-L-ArgMA was also estimated at 46.2 ± 8%, according to the difference between the surface below the two ^1^H NMR spectra of Poly-L-Arg and Poly-L-ArgMA (Fig. 1B).

### Characterization of PβAE and Poly-L-ArgMA/PβAE

**Fig. 2:**
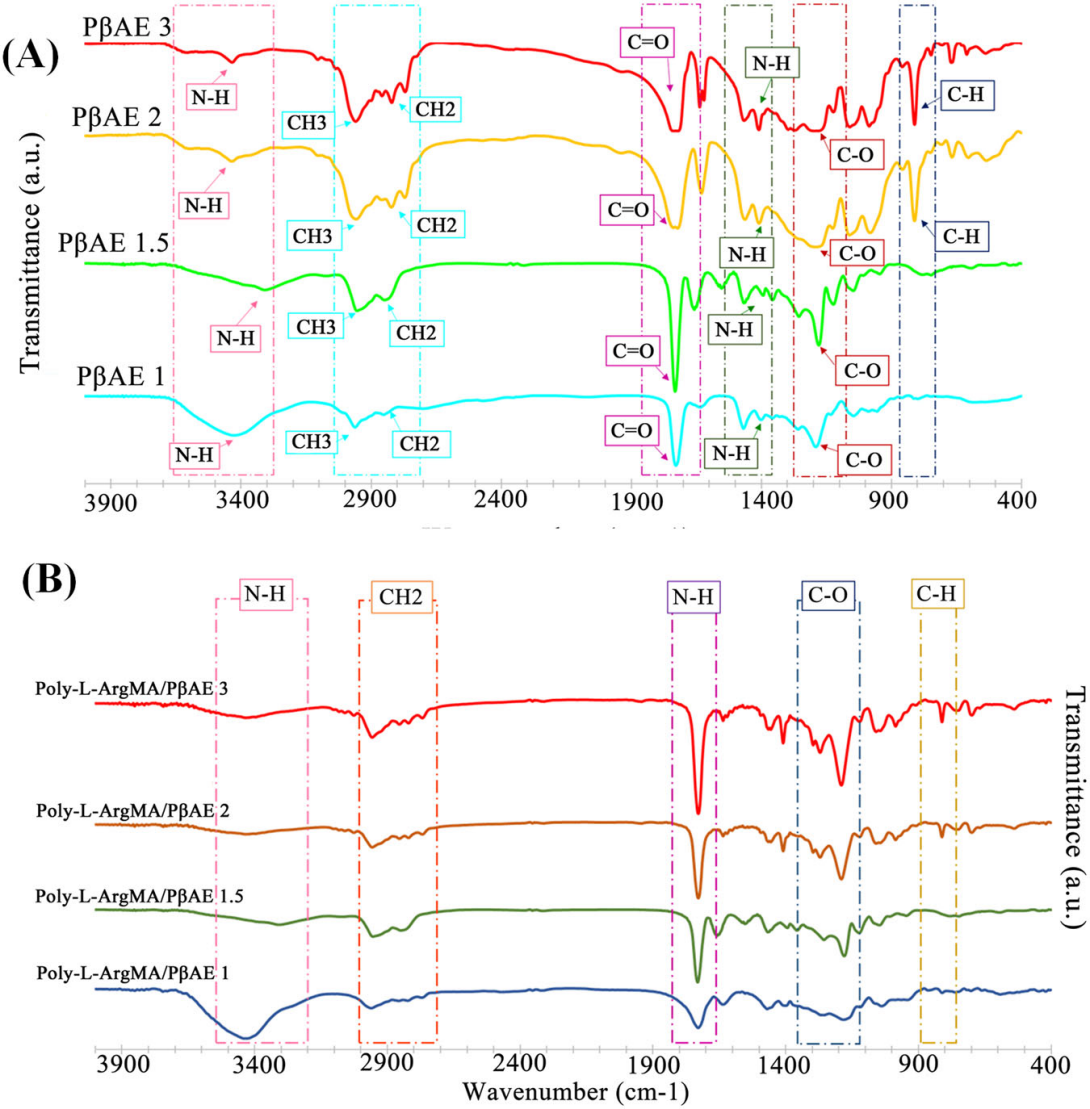
Chemical characterization of polymers and copolymers: FTIR spectra of (**A**) PβAEs, and (**B**) Poly-L-ArgMA/PβAEs copolymers

The biodegradable PβAE polymers were synthesized using four different molar ratios of diacrylate: diamine (1.1:1(PβAE1), 1.5:1(PβAE1.5), 2:1(PβAE2), and 3:1(PβAE3)) by Michael addition. The FTIR spectra of different PβAEs (Fig. 2A) consisted of some main characteristic peaks including C–O, C=O stretching, methyl absorption, and N–H bonds at 1191, 1722, 2962, 3421 cm^−1^, respectively, which indicated PβAE was successfully synthesized [19]. The difference between PβAEs was related to the intensity of peaks related to the acrylate part of the polymer, which was enhanced with increasing acrylate monomer content (Fig. 2A). The results demonstrated increasing the intensity of C–H bonds at 812 cm^−1^, C=O stretching bonds at 1730 cm^−1^, and CH_2_ bonds at 2820 cm^−1^ related to the acrylate group and decreasing the intensity of NH_2_ bonds at 3420 cm^−1^ by decreasing the molar ratio of diamine monomers in the PβAE1.5, PβAE2, and PβAE3 structures. The interaction between Poly-L-ArgMA and PβAEs was also confirmed by FTIR spectroscopy (Fig. 2B). After adding the Poly-L-ArgMA and EDC/NHS, the formation of anhydride and ester (~1730 cm^−1^) and also new peaks at 2700 cm^−1^ (CH), 1500 cm^−1^ (CN), 1300 cm^−1^ (C–O) were detected due to copolymerization of the polymers by using EDC/NHS [40].

### Characterization of Poly-L-ArgMA/PβAE

**Fig. 3:**
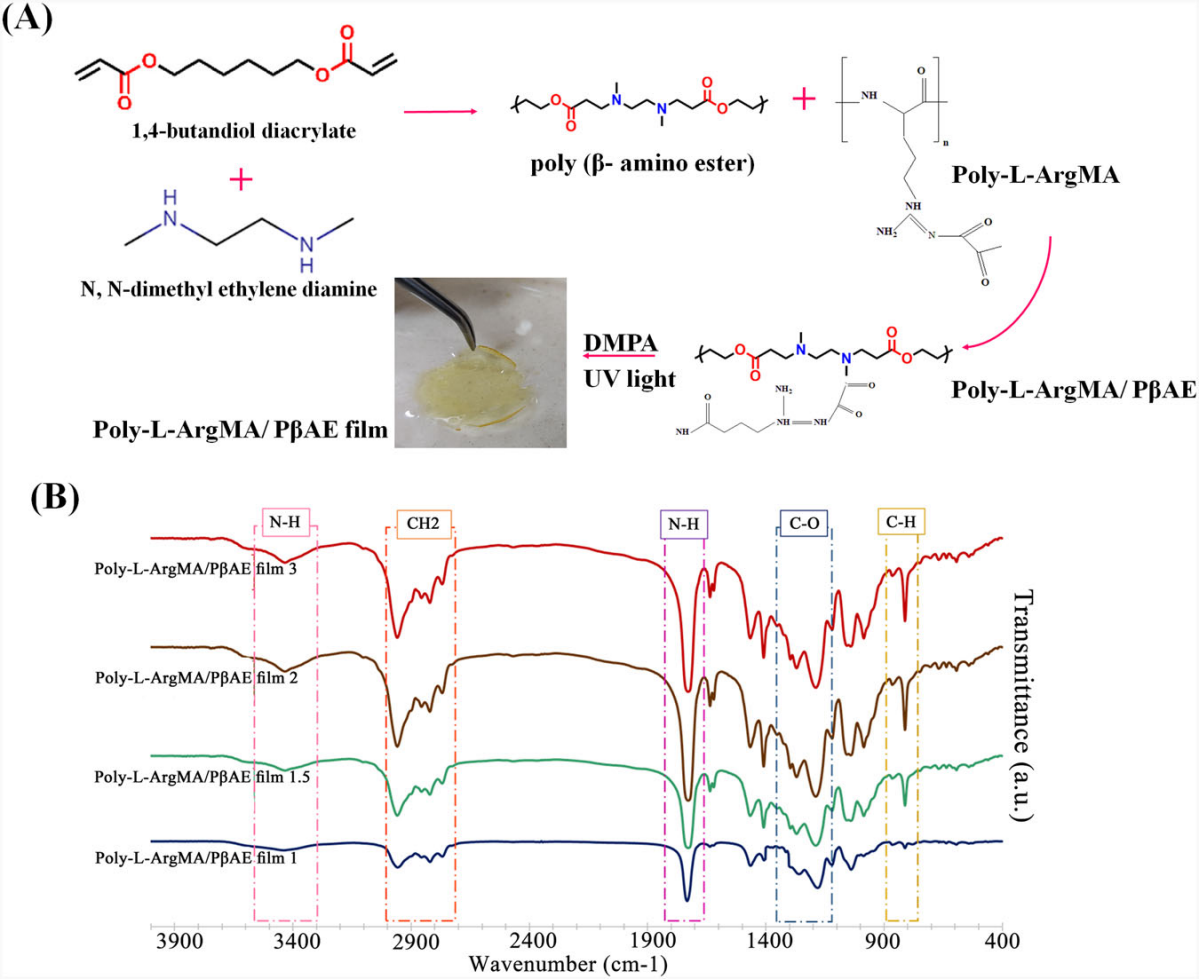
Chemical characterization of Poly-L-ArgMA/PβAEs hydrogel films: **A** The schematic showing the interaction of PβAE monomers and Poly-L-ArgMA/PβAEs (copolymerization) and synthesized UV-crosslinked Poly-L-ArgMA/PβAEs films, and **B** FTIR spectra of Poly-L-ArgMA/PβAEs films

Poly-L-ArgMA/PβAEs films were crosslinked under UV light using DMPA crosslinker. Figure 3A shows the schematic of the PβAE and Poly-L-ArgMA/PβAEs polymers and UV-crosslinked Poly-L-ArgMA/PβAEs hydrogels. The PβAE synthesized by the interaction between diacrylate and diamine monomers and its functional side chain groups (N–H and C=O) could bond with methacrylate chemical group in Poly-L-ArgMA. According to the spectrum of crosslinked films (Fig. 3B), when the DMPA was incorporated as a chemical crosslinker in the reaction of Poly-L-ArgMA/PβAE copolymer, the signal of C=O stretching vibrations appeared at 1728 cm^−1^ [41]. The comparison of this spectrum between Poly-L-ArgMA/PβAE copolymer without DMPA and Poly-L-ArgMA/PβAE copolymer with 1%wt DMPA can clearly see increasing in the intensity of peaks related to C–H, C–O, CH_2_ bonds at 812, 1188, 1400, and 2956 cm^−1^, respectively.

A simple method to control the molecular weight (MW) of polymers is to change the monomers molar ratio. In this study, the PβAEs were synthesized using a range of four monomer molar ratios between 1.1:1 and 3:1. According to Table 1, the molecular weight of these PβAEs increased from 2618 to 9100 Da by increasing diacrylate monomer ratio from 1.1 to 3. Similarly, Chen et al. [42] evaluated the effect of monomer molar ratio on PβAE physiochemical properties. Their results indicated that the monomers molar ratio is the essential parameters for adjusting the molecular weight of polymers and maximum molecular weight PβAE should be obtained by increasing acrylate monomers. On the other site, our results indicated that after the polymerization and methacrylation of L-Arg (Poly-L-ArgMA), the molecular weight increased from 117 to 1350 Da, which indicated the interaction between the monomer side chains and the successful polymerization. The molecular weight of Poly-L-ArgMA/PβAEs were evaluated and results demonstrated the molecular weight of all copolymers significantly increased compared to their polymers. For instance, the molecular weight of PβAE 1 and Poly-L-ArgMA/PβAEs 1 were 2618 and 3510 Da, respectively. These results indicated when the Poly-L-ArgMA and PβAE polymers were reacted with EDC/NHS the molecular weights enhanced with improving number of polymers chains entanglements.

**Table 1:** Mw polymer by gel permeation chromatography and zeta potential

Polymer	M_W_ (Da)	PDI	Zeta potential (mV)
L-Arg	117	0.980	+5.8 ± 1.1
Poly-L-Arg	1300	1.010	+9.9 ± 3.5
Poly-L-ArgMA	1350	1.130	+1.4 ± 0.6
PβAE 1	2618	1.298	+24.1 ± 3.8
PβAE 1.5	3140	1.340	+20.1 ± 0.8
PβAE 2	7150	1.584	+16.7 ± 4.1
PβAE 3	9100	1.765	+10.1 ± 0.6
Poly-L-ArgMA/PβAE 1	3510	1.432	+18.6 ± 4.7
Poly-L-ArgMA/PβAE 1.5	3980	1.443	+17.5 ± 3.1
Poly-L-ArgMA/PβAE 2	7945	1.604	+15.1 ± 1.8
Poly-L-ArgMA/PβAE 3	10510	2.060	+6.2 ± 1.1

To clarify the interaction between molecules, the zeta potential of polymers was determined. According to Table 1, PβAEs revealed a wide range of positive surface charges, depending on the acrylate monomer content. Noticeably, by increasing acrylate monomer in comparison with diamine monomers the surface charge of PβAE reduced from 24 mV (in PβAE1) to less than 10 mV (in PβAE3). This result was similarly reported in previous studies [43, 44]. Additionally, the positive surface charge of Poly-L-ArgMA/PβAEs reduced in the range of 18.6–6.2 mV in comparison with PβAEs. It might be due to increasing chemical interaction between amine and carboxylic polymers groups by adding EDC/NHS. The cell/biomaterials interactions is driven by the zeta potential of biomaterials [45]. In general, due to the negative surface charge of mammalian cells, relative positive zeta potential of biomaterials could improve their interactions with cells. Schulz et al. [46] studied the role of surface charge of Poly(amidoamine)-alginate hydrogel on the mesenchymal stem cell responses and found that positive surface charge of hydrogels enhanced the cell spreading and the expression of adhesion gene integrin.

### In vitro physiological stability evaluation of Poly-L-ArgMA/PβAE

**Fig. 4:**
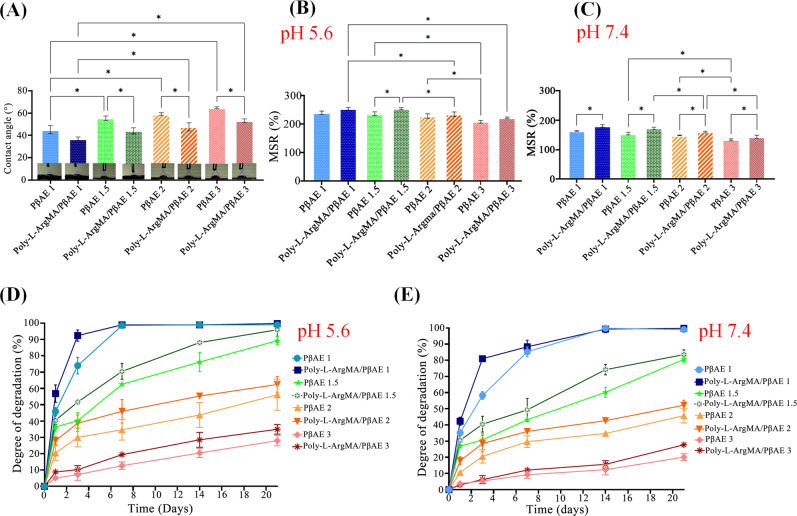
The physiological stability of Poly-L-ArgMA/PβAE and PβAE films: **A** The wettability of PβAE and Poly-L-ArgMA/PβAE films, the mass swelling ratio of films after 24 h in PBS (**B**) at pH 5.6, (**C**) at pH 7.4, The weight loss of films during 21 days-soaking in PBS (**D**) at pH 5.6, and (**E**) at pH 7.4. All values are characterized as corresponding to the averages (*n* = 3) ±standard deviation. (*P < 0.05)

The wettability of Poly-L-ArgMA/PβAE films in different groups was analyzed using water contact angle measurement (Fig. 4A). Results showed that the water contact angle of PβAE samples was significantly adjustable with monomers ratio changes (*P* < 0.05). Noticeably, water contact angle enhanced from 45.2 ± 8° to 64 ± 2° with increasing acrylate monomer from 1.1 to 3. By reducing the amino hydrophilic monomers ratio in comparison with acrylic monomer, the interaction between the polymer chains and water molecules reduced, as similarly reported in other studies [42, 47]. On the other hand, after the incorporation of hydrophilic Poly-L-ArgMA, the water contact angle significantly decreased (*p* < 0.05). Noticeably, the water contact angle of PβAE1 reduced from 45.2 ± 6° to 32 ± 3°, after copolymerization process. It should be noted the increased hydrophilicity of samples by using Poly-L-ArgMA which might have an essential role in cell responses, such as cellular adhesion and proliferation [48].

One of the crucial factors in biomaterials applied in tissue engineering is water absorption ability, and degradation rate in biological environments. pH-responsive polymers are considered among researchers due to their ability to modulate their swelling ratios in various pH environments making them attractive for tissue engineering and drug delivery applications [49]. To confirm the pH-sensitivity of our polymers, their swelling ratio was investigated at two different environments having various pH values (5.6 and 7.4). According to Fig. 4B, C, the hydrophilic polymer (Poly-L-ArgMA), the monomer molar ratio, and the pH of environment considerably modulated the swelling ratio of polymers. Noticeably, the swelling ratio of Poly-L-ArgMA/PβAE1 at pH 5.6 was 240 ± 10%, which was significantly higher than that of this value at pH 7.4 (163.5 ± 7%). It might be due to the cationic nature of PβAE [50]. According to the wettability results, the PβAE1 and its copolymer were also more hydrophilic than other groups, which resulted in enhanced water absorption. The high swelling ratio of PβAE1 and its copolymers could be due to hydrophilic diamine units, which could be easily interacted with water molecules. Similar result was reported by Vuk et al. [51] on PβAE based hydrogel with different acrylate and diamine monomers. They showed that increased amine groups in the structure could improve water molecule penetration in the polymer network and consequently improved swelling ratio. Additionally, the copolymerization of Poly-L-ArgMA/PβAEs enhanced the swelling ratio of hydrogels at pH~7.4 as shown in Fig. 4C. It might be due to the presence of hydrophilic Poly-L-ArgMA which enhanced the interaction between hydrogel and water molecules, leading to improved swelling ratio.

**Fig. 5:**
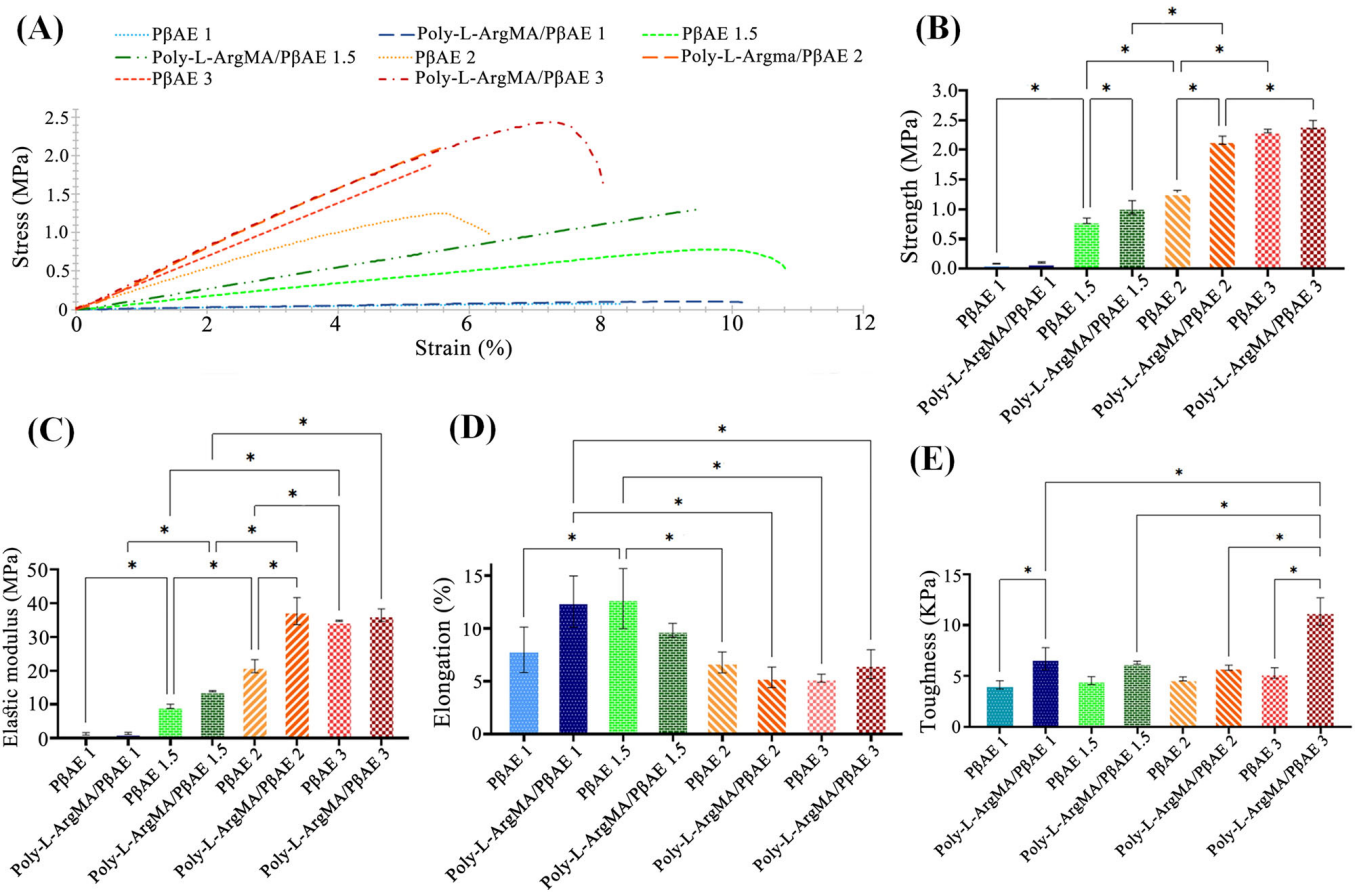
Mechanical characteristics of Poly-L-ArgMA/PβAE and PβAE films: **A** Stress-strain curves, **B** tensile strength, **C** elastic modulus, **D** elongation, and **E** toughness of hydrogels. All values are characterized as corresponding to the averages (*n* = 3) ±standard deviation. (*P < 0.05)

The weight loss of Poly-L-ArgMA/PβAE hydrogels was also evaluated at pH 5.6 and 7.4 (Fig. 4D, E). Our results showed that the degradation rate of samples significantly adjusted with monomer molar ratio. For instance, the weight loss of Poly-L-ArgMA/PβAE reduced from 99 ± 1%, to 27.98 ± 3.2% when the monomer molar ratio changed from 1.1:1 (PβAE 1 sample) to 3:1 (PβAE 3), after 21 days’ immersion at pH 5.6 (Fig. 5A). Similarly, Altuncu et al. [52] studied degradation rate of novel PβAE gels with wide range of monomers molar ratio. Their results indicated that the degradation rate depended on PβAE chemical structure and more hydrophilic PβAE gel has more water uptake, more cleavage of ester linkages and faster degrade rate than hydrophobic gel. Additionally, our results showed the Poly-L-ArgMA/PβAE hydrogels degraded faster than PβAE films. For instance, the degradation rate of PβAE1.5 and Poly-L-ArgMA/PβAE1.5 samples were estimated 76.2 ± 5.9% and 88.1 ± 1.9%, at pH 5.6 during 14 days, respectively (*p* < 0.05). The fast degradation rate of Poly-L-ArgMA/PβAE films was due to the higher swelling ratio than PβAE films. As shown in Fig. 4D, E, both PβAE and Poly-L-ArgMA/PβAE degraded faster at pH 5.6 than at pH 7.4. For instance, the degradation rate of Poly-L-ArgMA/PβAE1.5 was measured 95.9 ± 4.2 and 83.5 ± 3% after 21 days’ immersion at pH 5.6 and 7.4, respectively (*p* < 0.05). These results confirmed that the decreasing pH environment enhanced the polymers hydrolysis by facilitating water absorption [53]. The above results indicated that the PβAE and Poly-L-ArgMA/PβAE degradation process depended on adjusting monomer molar ratio, copolymerization and pH environment. The controlled degradation of pH-sensitive PβAE and Poly-L-ArgMA/PβAE might be ideal for various biomedical applications especially in soft tissue engineering and drug delivery. According to previous studies, the degradation rate of scaffolds should be equal to the rate of soft tissue formation [54]. According to previous studies, the faster degradation profile of Poly-L-ArgMA/PβAE 1 and Poly-L-ArgMA/PβAE1.5 (70–100% during 14 days) made them suitable for acute and chronic wound healing application [55]. In another word, the slow degradation of Poly-L-ArgMA/PβAE2 and Poly-L-ArgMA/PβAE3 (20–30% during 21 days) made it suitable for vascular tissue engineering and formation new blood vessels [56].

### Mechanical properties of Poly-L-ArgMA/PβAE films

The role of Poly-L-ArgMA and different PβAE monomers molar ratio on the mechanical performances were investigated using tensile test. The stress-strain curves of the films are presented in Fig. 5A. Results demonstrated that increasing acrylate monomers considerably promoted the tensile strength of PβAE and Poly-L-ArgMA/PβAE, while the elongation decreased significantly. According to the stress-strain curves, the tensile strength, elastic modulus, elongation, and toughness were measured and presented in Fig. 5B–E, respectively. Results demonstrated that the strength of Poly-L-ArgMA/PβAE1 (0.10 ± 0.04 MPa) was significantly enhanced with increasing acrylate monomer to 2.42 ± 0.3 MPa at Poly-L-ArgMA/PβAE3 sample (*P* < 0.05). A similar trend was reported for PβAE films. Similarly, Tamer et al. [57] evaluated the mechanical properties of PβAE with two different monomer molar ratios (1.4:1 and 1.5:1 acrylate: amine) and showed with increasing acrylate monomer, the strength enhanced from 0.6 MPa to 1 MPa. Additionally, the results indicated that the tensile strength of Poly-L-ArgMA/PβAE compared to pure PβAE. It might be due to strong chemical bonding and interaction between carboxylic groups in DMPA as a UV-crossliker and methacrylate/amine chemical groups of Poly-L-ArgMA/PβAE copolymers [58]. The elastic modulus (Fig. 5C) revealed a similar trend with tensile strength of hydrogels. For instance, the elastic modulus enhanced from 21.3 ± 4 MPa to 37.7 ± 7 MPa, after copolymerization of PβAE2 with Poly-L-ArgMA. Similarly, Brey et al. [59] evaluated chemical and mechanical properties of photo-crosslinkable PβAE with different monomers ratios. Their studies showed with increasing acrylate monomer from 1.1 to 1.4, the elastic modulus increased from 0.22 ± 0.012 MPa to 4.50 ± 1.08 MPa. According to Fig. 5D, the elongation of UV-crosslinked hydrogels was considerably improved with decreasing diacrylate monomer molar ratio from 3 to 1.5 and 1.1. Additionally, the toughness value of hydrogels was calculated from the stress-strain curves (Fig. 5E). Results indicated that the toughness improved with increasing acrylate monomer molar ratio from 1.1 to 3 and adding Poly-L-ArgMA to PβAE polymer. Although Poly-L-ArgMA/PβAE3 showed the highest elastic modulus, tensile strength and toughness, they did not match with the mechanical properties of soft tissues. Consequently, Poly-L-ArgMA/PβAE1.5 showing similar mechanical properties to the natural tissues are more favorable for soft tissue engineering, specially skin tissue engineering.

**Fig. 6:**
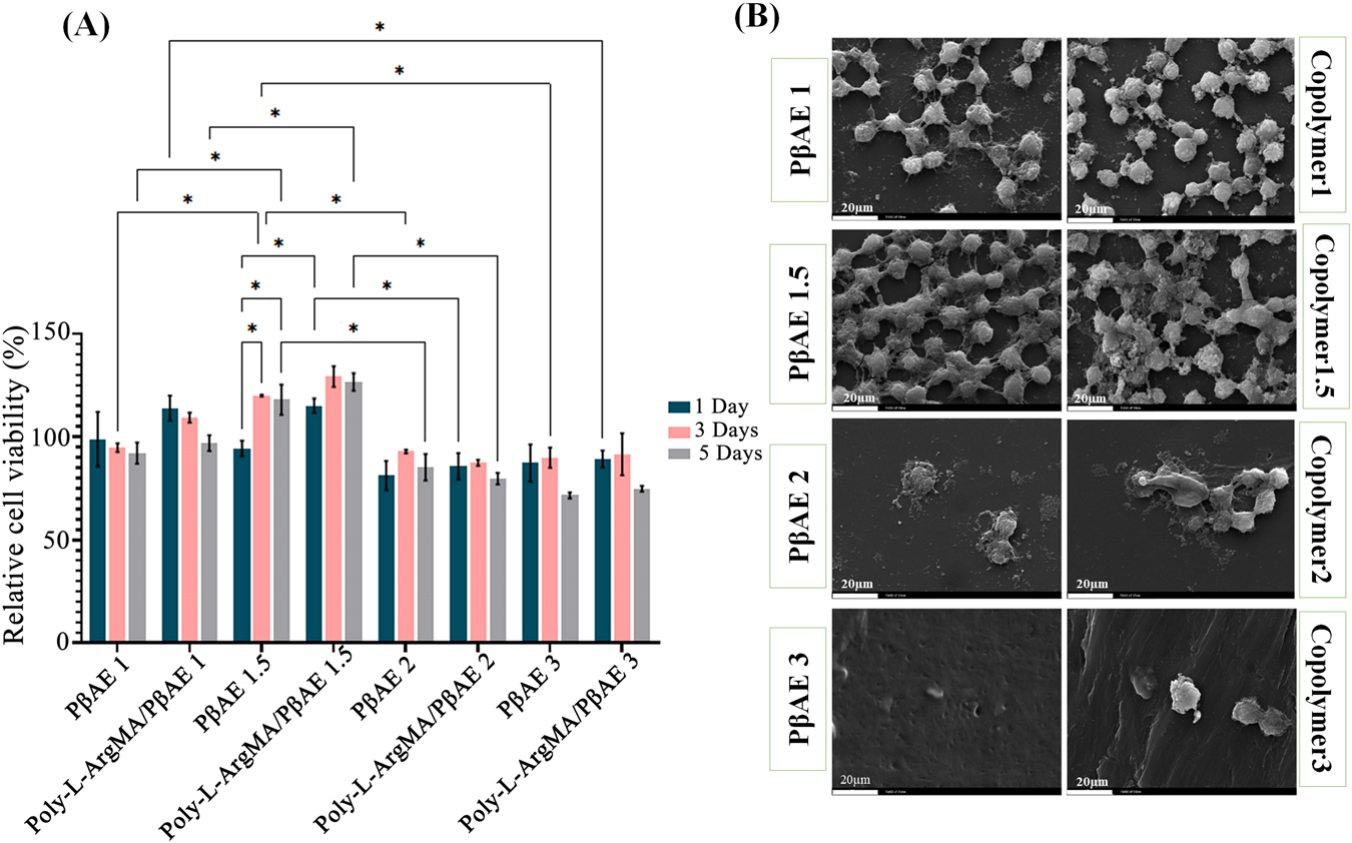
**A** Relative fibroblast cell viability in various samples measured using the MTT test for 5 days and normalized against the control (TCP), and **B** The SEM images of L929 cells adhered to the surface of PβAE and Poly-L-ArgMA/PβAE films, 3 days after cell seeding. All values are described as corresponding to the averages (*n* = 3) ±standard deviation. (*P < 0.05)

### Cell culture

In vitro biocompatibility of the Poly-L-ArgMA/PβAE hydrogels was evaluated according to the cell viability, proliferation and attachment assays (Fig. 6). MTT assay was used to determine the viability of different samples, after 1, 3 and 5 days of culture (Fig. 6A). The cell viability results demonstrated the positive effect of Poly-L-ArgMA on L929 cell proliferation. Furthermore, among the PβAE with different monomers molar ratio, the results indicated that the L929 cells had better viability in the less acrylate monomer molar ratio in comparison with diamine monomer (1.1:1 and 1.5:1 acrylate: diamine molar ratio) than the higher ones (2:1 and 3:1). For instance, the cell viability in contact with Poly-L-ArgMA/PβAE1, Poly-L-ArgMA/PβAE1.5, Poly-L-ArgMA/PβAE2 and Poly-L-ArgMA/PβAE3 was estimated 97 ± 5 (%control), 126 ± 7 (%control), 80 ± 4 (%control) and 74 ± 2 (%control), after 5 days, respectively. The excellent cell performances in the samples containing less acrylate monomers was related to their hydrophilic surface and appropriate mechanical performances, as similarly reported before [60]. Lin et al. [61] evaluated the cell viability in contact with poly(γ-glutamic acid) (γ-PGA). Their studies demonstrated that the hydrogel had an excellent cell viability with increasing hydrophilicity and mechanical properties, mimicking the surrounding soft tissue.

Figure 6B shows the SEM images of the cells attached on the samples. The significant cell attachment and anchoring to samples confirmed the improved cell responses in contact with Poly-L-ArgMA/PβAE hydrogels. The hydrogels with minimum PβAE acrylate monomer (1.1:1 and 1.5:1 acrylate: diamine) created a suitable environment for fibroblasts enhancing their attachment, migration and proliferation. These results are consistent with those published by Yao et al. [62], where the number of Schwann cells on Poly(β-amino ester)-based hydrogels with 0.025 MPa strength was significantly higher than that on 0.042 MPa. In this study, fibroblasts spread on the PβAE1.5 and its copolymer, whereas the cells agglomerated and formed aggregates without spreading on the surface of PβAE1 and its copolymers. In general, there are three types of cell and polymer interactions [63]. The first type is a non-adhesion interaction when cells are unable to adhere to a biomaterial surface. Secondly, passive adhesion occurs when cells interact and adhere, but cannot easily spreading and agglomerated on the surface. The final interaction is an active adhesion interaction, in which cells spontaneously interact and stably speared to the polymer surface [64, 65].

In this study, the cell adhesion process for PβAE1.5 and its copolymer films show active adhesion between the cells and the surface of the films. This can be due to the suitable hydrophilicity, the surface charge and chemical structure of PBAE1.5 to interact with fibroblast cells [66].

As mentioned the PβAE1.5 and Poly-L-ArgMA/PβAE1.5 have proper mechanical properties, cell viability and cell attachment ability for different soft tissue engineering.

### In vitro antibacterial activity

**Fig. 7:**
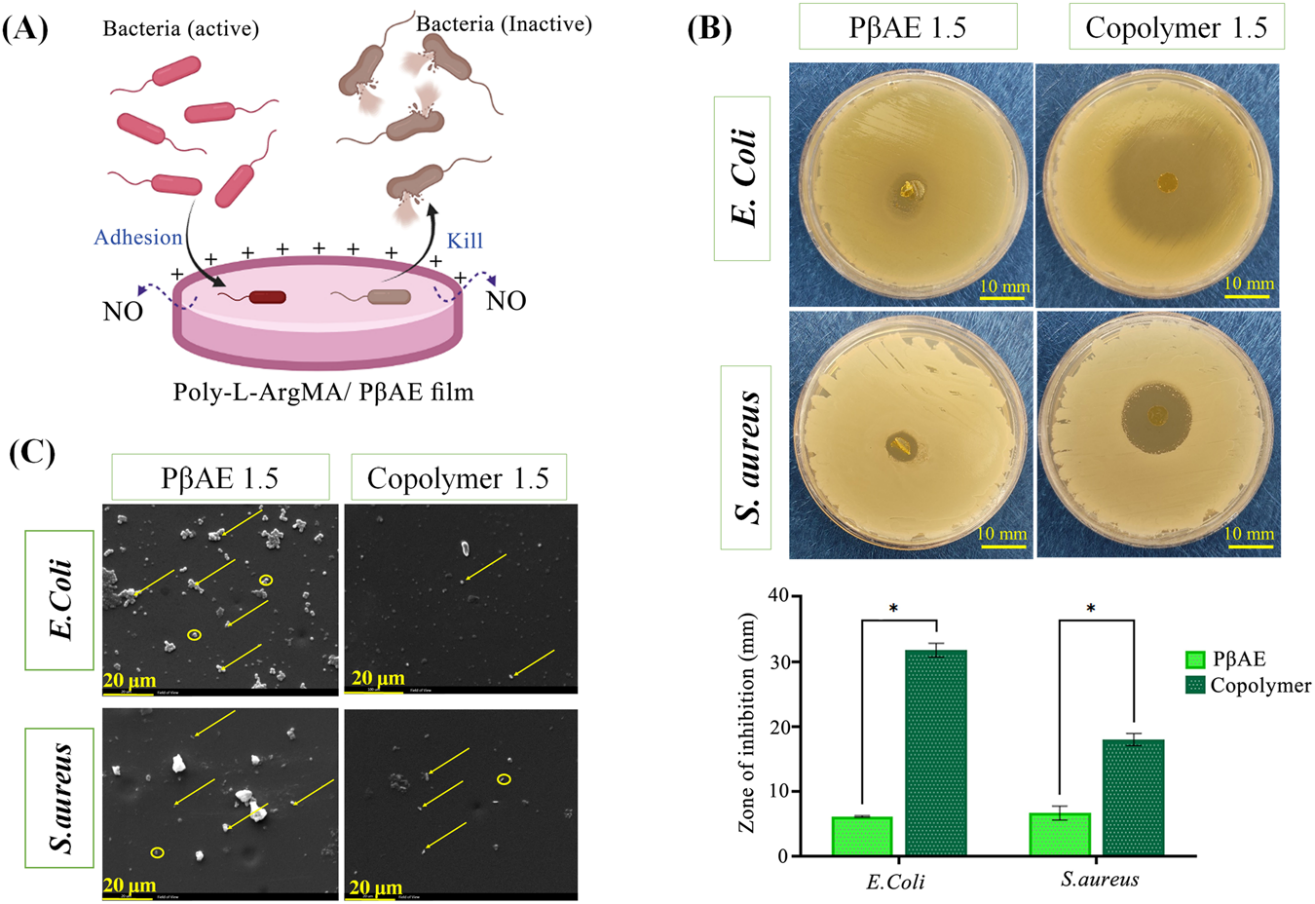
**A** The schematic of Poly-L-ArgMA/PβAE antibacterial activity, **B** zone of inhibition bar graph of Poly-L-ArgMA/PβAE1.5 and PβAE 1.5 films against *E. coli* and *S.aureus*, and **C** bacteria adhesion images of *S. aureus* and *E. coli* on the surface of hydrogels. All values are described as corresponding to the averages (*n* = 3) ±standard deviation. (*P < 0.05)

The infection is a challenge in tissue engineering, which may postpone the regeneration process of damaged tissues [67]. In this study, the antibacterial properties of PβAE1.5 and Poly-L-ArgMA/PβAE1.5 was examined according to the zone-inhibition method against both *S. aureus* and *E. coli* bacteria (Fig. 7). As shown in Fig. 7A, Poly-L-ArgMA/PβAE exhibited antibacterial activity because of the protonated amino groups, positive surface charge and oxidative pathway (production of nitric oxide) on the surface of hydrogels, which could kill Gram-negative and Gram- positive bacteria through damaging bacteria cell wall. Additionally, as shown in Fig. 7B, Poly-L-ArgMA/PβAE showed greater inhibition zone than pure PβAE, against both bacteria, due to the providing of oxidative pathway and NO production in the presence of Poly-L-Arg. For instance, the inhibition zone of pure PβAE and Poly-L-ArgMA/PβAE against *E.Coli* were 6.1 ± 0.5 mm and 31.8 ± 2.5 mm, respectively (*p* ˂ 0.05). Similarly, Su et al. [68] found that the antibacterial properties of chitosan was significantly improved after modification with arginine. Additionally, the different antibacterial activity between *S.aureus* and *E.coli* could be due to bacterial various structures [69]. Gram-positive bacteria such as *S.aureus* have a compact cell wall with interconnecting peptidoglycan layers. However, the Gram- negative bacteria like *E.Coli* have a porous lipid bilayer cell wall. Overall, the Poly-L-ArgMA/PβAE 1.5 hydrogels could significantly enhance antibacterial properties, which is promising for accelerating soft tissue regeneration.

To further visualize the bacterial interaction with hydrogels, the adhesion of *E.Coli* and *S. aureus* was evaluated on the PβAE and copolymer hydrogels. According to Fig. 7C, after 24 h of bacteria incubation, the surface morphologies of the hydrogels were observed using SEM. The number of rod-shaped *E.Coli* bacteria adhered on the poly-L-ArgMA/PβAE hydrogels was appreciably reduced compared to the pure PβAE sample. Similarly, poly-L-ArgMA/PβAE hydrogels effectively lowered the amounts of spherical Gram-positive *S.aureus* bacteria adhesion. Bacterial adhesion is the most critical phase of bacterial colonization and infection on wound site [70]. The initial adhesion of microorganisms depends on the overall physicochemical characteristics of the microbial cell surface, the biomaterial surface and the biological properties [71]. In the present study, by adding poly-L-ArgMA to hydrogel the inhibition of *E.Coli* and *S.aureus* growth on the hydrogel surface were promoted, which could reduce infections in soft tissue remodeling specifically wound healing applications.

Our results revealed that the PβAE with diacrylate to diamine monomers molar ratio of 1.5:1 could be suitable candidate for soft tissue engineering, according to physical and mechanical properties. Moreover, the incorporation of Poly-L-ArgMA to structure of PβAE1.5 hydrogels resulted in improved cell viability, enhanced cell spreading, and promoted antibacterial activation. Despite these strengths, our study has several distinct limitations, including the prolonged methacrylation process and low copolymerization efficiency. Although the physical and biological results showed Poly-L-ArgMA/PβAE is suitable for soft tissue engineering, especially wound healing applications, still they need to be analyzed in vivo to confirm their applicability.

## Conclusion

In summary, we successfully synthesized and evaluated a series of pH-sensitive and antibacterial hydrogels based on Poly-L-ArgMA/PβAE with a wide range of chemical and mechanical properties, antibacterial activity, and biocompatibility for soft tissue engineering. The hydrogels could be fabricated via the polymerization of L-Arg, the methacrylation of Poly-L-Arg, copolymerization of Poly-L-ArgMA with PβAE and finally UV-crosslinking process. The obtained hydrogels showed different swelling ratios according to the chemical structure and the environmental pH. Furthermore, the results demonstrated that the PβAE monomers molar ratio, Poly-L-ArgMA content, and environmental pH were critical parameters to control degradation rate and mechanical properties. Interestingly, compared to the pure PβAE, the Poly-L-ArgMA/PβAE copolymer films improved cell viability, and adhesion, while enhance the antibacterial properties against both Gram-positive and Gram-negative bacteria. Overall, the pH-sensitive and antibacterial Poly-L-ArgMA/PβAE hydrogels with different PβAE monomer ratios could provide suitable physical-mechanical properties, and biological activity for various tissue engineering applications. Specifically, Poly-L-ArgMA/PβA1.5 is a suitable choice for soft tissue engineering, specifically wound healing applications.
